# Supplementary material for: Polyzwitterionic Coating of Porous Adsorbents for Therapeutic Apheresis
Source: J Funct Biomater. 2022 Nov 3;13(4):216. doi: 10.3390/jfb13040216 (PMC9680258; doi:10.3390/jfb13040216)
Supplement: Supplementary file 1 [file jfb-13-00216-s001.zip › SI_jfb-1995511.pdf]

Supplementary Information (SI)

Polyzwitterionic Coating of Porous Adsorbents for Therapeutic Apheresis

Vladislav Semak, Tanja Eichhorn, René Weiss, Viktoria Weber*

Department for Biomedical Research, Center for Biomedical Technology,
University for Continuing Education Krems, 3500 Krems, Austria

* Correspondence: *vladislav.semak@donau-uni.ac.at*

Table of Contents

1 Materials and Reagents 3

2 Methods 6

3 Experimental 9

3.1 Synthesis of the Monomers 9

3.1.1 Synthesis of *N*-(4-azido)-2-methylacrylamide (Az-MAAm) 9

3.1.2 Synthesis of 2-((7-nitrobenzofuraz-4-yl)amino)ethyl methacrylate (NBD-MA) 10

3.1.3 Synthesis of Carboxybetaine (CBMA) Monomers with Methylene (C1) and Pentylene (C5) Spacers 11

3.2 Synthesis of the Zwitterionic Copolymers 12

3.2.1 Synthesis of Sulfobetaine Copolymer pSBE, *i.e.* poly[SBE-*co*-Az-*co*-NBD] 12

3.2.2 Synthesis of Carboxybetaine Copolymer pCBMA-C1, *i.e.* poly[CBMA-C1-*co*-Az-*co*-NBD]. 13

3.2.3 Synthesis of Carboxybetaine Copolymer pCBMA-C5, *i.e.* poly[CBMA-C5-*co*-Az-*co*-NBD]. 14

4 Quantitative Analysis of 4-Azido Aniline Moiety in poly[SBE-*co*-Az] Bipolymer by UV/Vis Spectrophotometry 16

5 4-Azidoaniline Hydrochloride Photolysis (FTIR Kinetic Study) 18

6 Evaluation of Coating Stability 20

7 Energy-Dispersive X-ray Spectroscopy Analysis 22

8 Evaluation of Polymer-Induced Hemolysis 23

9 Recirculation of Whole Blood over DALI Columns 24

10 Scanning Electron Microscopy (SEM) Micrographs of Adsorbents 25

11 Tables of the Most Important FTIR Absorption Bands 26

12 Abbreviations 31

13 References 32

1 Materials and Reagents

Chemicals. *N*-(3-(dimethylamino)propyl)methacrylamide [CAS Number 5205-93-6], methyl bromoacetate [CAS Number 96-32-2], ethyl 6-bromohexanoate [CAS Number: 25542-62-5], 4-chloro-7-nitrobenzofurazan [CAS Number 10199-89-0], ethanolamine [CAS Number 141-43-5], methacryloyl chloride [CAS Number 920-46-7], 2,2′-azobis(2-methylpropionitrile) (AIBN) 0.2 M solution in toluene [CAS Number 78-67-1], [2-(methacryloyloxy)ethyl]dimethyl-(3-sulfopropyl)ammonium hydroxide [CAS Number 3637-26-1] were acquired from Sigma-Aldrich (*i.e.* Merck, Darmstadt, Germany) and Amberlite® IRA-910 (chloride form) from Dow Chemicals (Midland, MI). Unfractionated heparin was purchased from Gilvasan Pharma (Vienna, Austria).

4-Azidoaniline was prepared from 4-iodoaniline with sodium azide under Cu_2O/tetraethylammonium prolinate catalysis according to published protocol [1]. Alternatively, the corresponding hydrochloride salt [CAS Number: 91159-79-4] from Sigma-Aldrich was used. Organic solvents and inorganic salts were obtained from Carl Roth. All chemicals were used as received except for methacryloyl chloride [CAS Number 920-46-7] which was distilled before use [2].

Buffers & Priming Solutions. Phosphate buffered saline (PBS) was from Thermo Fisher Scientific (Waltham, MA), and glutaraldehyde was obtained from Carl Roth (Karlsruhe, Germany). DALI adsorbent priming solution (134 mM Na^+, 4 mM K^+, 1.75 mM Ca^{2+}, 0.5 mM Mg^{2+}, 106.5 mM Cl^-, 36 mM HCO_3^-) and ACD-A (22.0 g/L trisodium citrate dihydrate, 24.5 g/L glucose monohydrate, 7.3 g/L citric acid) were obtained from Fresenius Medical Care (Bad Homburg, Germany).

Reagents for the Determination of Coagulation Parameters

Prothrombin Time	Siemens Healthineers, Dade® Innovin, Ref: B4212-50
Activated Prothrombin Time	Siemens Healthineers, Dade ® Actin FS, Ref: B4218-20
Thrombin Time	Siemens Healthineers, Test Thrombin Reagent, Ref: OWHM 13
Fibrinogen	Siemens Healthineers, Dade® Thrombin Reagent, Ref: B4233-25
Antithrombin III	Siemens Healthineers, THR Substrate, Ref: OWWR17
Protein C	Siemens Healthineers, Activator and Substrate, Ref: OUVV 17
Control Plasma	Siemens Healthineers, normal, Ref: 10484201

All measurements were performed on a coagulation analyzer Sysmex CA-560 (Siemens Healthineers, Erlangen, Germany).

Reagents for the Determination of Lipoprotein(a), Low and High Density Lipoprotein Cholesterol

LDL_C	LDL-Cholesterol plus 2nd gen., ID: 07 6627 5 (Cobas®, Roche Diagnostics)
HDLC3	HDL-Cholesterol plus 3rd gen., ID: 07 6833 2 (Cobas®, Roche Diagnostics)
LPA2	Tina-quant Lipoprotein(a) 2nd gen., ID: 07 7504 5 (Cobas®, Roche Diagnostics)

All measurements were performed on an automated analyzer Cobas c311 (Roche Diagnostics, Rotkreuz, Switzerland).

Human Whole Blood. Whole blood was collected by aseptic venipuncture from healthy volunteer donors (as approved by the Ethical Review Board of University for Continuing Education Krems) into vacutainer tubes (Vacuette Blood Collection Tubes, Greiner Bio-One, Kremsmuenster, Austria) containing ethylene diamine tetraacetic acid (EDTA), sodium heparin, or sodium citrate as anticoagulant. Written informed consent was obtained from all donors. For flow experiments, aliquots of 50 mL of venous blood were drawn from healthy adult volunteers into medical grade blood bags (Fresenius Medical

Care) containing ACD-A and heparin to obtain final concentrations of ACD-A 1:20 (5.6 mM citrate) supplemented with 0.8 IU/mL heparin. Before the experiments were completed, blood count (Sysmex KX-21 N, Sysmex, Neumuenster, Germany) was performed from EDTA anticoagulated whole blood.

2 Methods

Flow Cytometric Characterization of Platelets and Extracellular Vesicles (EVs). Platelets and EVs were characterized by flow cytometry using a Gallios Flow Cytometer (Beckman Coulter, Brea, CA). For staining, samples were diluted 1:100 in sterile-filtered PBS (0.1 μm Minisart syringe filter, Sartorius Stedim Biotech, Goettingen, Germany). To characterize platelets, aliquots (100 μl each) of the diluted samples were incubated for 15 min at room temperature in the dark with a combination of PC7-conjugated anti-CD41 (Beckman Coulter) as platelet marker and FITC-conjugated anti-CD62P (Beckman Coulter) as platelet activation marker [3]. To characterize EVs, diluted samples were stained with FITC-conjugated lactadherin (Haematologic Technologies, Essex Junction, VT) as marker for EVs exposing phosphatidylserine, PC7-conjugated anti CD41, and with APC-conjugated anti-CD235a as red blood cell marker (eBioscience, San Diego, CA). To remove any precipitates, fluorochrome conjugates were centrifuged at 18,000 x g for 10 min at 4°C prior to use. Calibration of the flow cytometer was performed with fluorescent silica beads (1 μm, 0.5 μm, 0.3 μm; excitation/emission 485/510 nm; Kisker Biotech, Steinfurt, Germany). The triggering signal was set on the forward scatter/size, and the EV gate was defined as previously described [4–6]. Prior to analysis, stained samples were diluted 1:5 in sterile-filtered PBS. Platelets were identified as $CD41^+$ cells, and EVs were identified as $lactadherin^+$ events in the EV gate. Acquisition was performed for 3 min at a flow rate of 10 μl/min. Data were analyzed using the Kaluza Analysis Software 2.1 (Beckman Coulter).

Fluorescent and Confocal Laser Scanning Microscopy. To obtain thin sections of the adsorbent beads, adsorbents were mixed with optimal cutting temperature compound (Tissue-Tek OCT Compound, Sakura Finetek Europe, Alphen aan den Rijn, Netherlands) at room temperature. A dense suspension was placed onto a plastic mold, frozen, and 10

μm slices were cut using a cryostat (CryoStar™ NX70 Cryostat, Thermo Scientific). The thin sections were transferred onto adhesion slides (HistoBond adhesive microscope slides, Marienfeld, Germany), and mounted with Fluoromount aqueous mounting medium (Sigma Aldrich). Fluorescent images were acquired at excitation wavelengths of 488 nm (green FITC laser line) using a fluorescent (BX51 Olympus, Tokyo, Japan) and confocal laser scanning microscope (TCS SP8 Leica, Mannheim, Germany) equipped with 2x, 4x and 10x objectives. Image analysis was performed using the CellR (Olympus) and LAS X software (Leica).

Scanning Electron Microscopy (SEM). Adsorbent beads were dehydrated using an ethanol gradient (0% to 96%), air-dried for 12 h at room temperature, sputter-coated with gold (Q150R ES, Quorum Technologies, Laughton, UK), and analyzed by scanning electron microscopy (TM-1000 Hitachi, Tokyo, Japan).

Energy-Dispersive X-ray Spectroscopy (EDX). The presence of polyzwitterion coatings on the adsorbent beads was assessed with the energy dispersive X-ray spectroscopy (EDX) function of the scanning electron microscope (TM-1000 Hitachi) on dried beads without gold sputtering. The key elements identified in EDX spectra included C (0.277 keV), N (0.392 keV), O (0.525 keV) and S (2.306 and 0.149 keV). In some cases, Na (1.039 keV) and Cl (2.622 and 0.182 keV) derived from the saline solution used for sample storage were detected, as well.

Fourier-Transform Infrared Spectroscopy (FTIR). All measurements were performed on Spectrum Two FT-IR Spectrometer (PerkinElmer, Waltham, MA) equipped with a $LiTaO_3$ detector and with a MIRacle single reflection Zinc Selenide ATR (ZnSe) accessory (PIKE Technologies, Madison, WI). Full FTIR spectra (650 to 4000 cm^{-1}) were recorded with 16-

64 scans at a nominal resolution of 2-4 cm^{-1}. For spectral manipulation Spectrum 10 (PerkinElmer) and Spectragryph 1.2.15 (Dr. Friedrich Menges, Oberstdorf, Germany) software packages were used.

Statistical Analysis. Statistical analysis and graphical visualization were performed using GraphPad Prism 9.3.1 (GraphPad Software, San Diego, CA). Statistics were performed using one-way ANOVA and Tukey´s multiple comparisons tests. For comparison of two groups of normally distributed data, paired t-test or unpaired t-test was used. Data are presented as means ± standard deviation (SD). A p-value ≤ 0.05 was considered as statistically significant.

Graphics software. Chemical structures and reaction schemes were drawn by the ChemDraw (PerkinElmer Informatics). For creation of figures Biorender and Inkscape graphics software were used. A green bead in the graphical abstract was created by DALL·E 2 (artificial intelligence system).

3 Experimental

For polymerization reactions, Schlenk tubes DURAN® with total volume of 50 mL and conical ground joint 14/23 were employed. Reaction mixtures were heated in an oil bath and the external temperature was monitored. Dialysis Spectra/Por® 3 cellulose tubing with MWCO 3500 from Spectrum Chemical (Gardena, CA) was used. Chromatography refers to flash column chromatography and was carried out on SiO_2 (silica gel 60, 230-400 mesh) from Carl Roth (Karlsruhe, Germany). Synthetized compounds were characterized in detail by FTIR and most important absorption bands are included in the chapter No. 11. Purity of synthetized monomers was verified before the set-up of polymerization reaction by thin-layer chromatography (TLC).

3.1 Synthesis of the Monomers

3.1.1 Synthesis of *N*-(4-azido)-2-methylacrylamide (Az-MAAm)

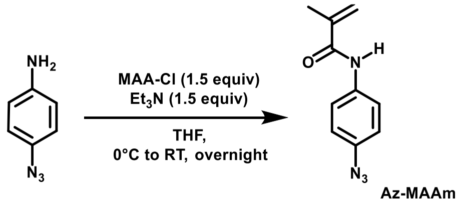

Triethylamine (95 µL, 0.67 mmol, 1.5 equiv) was added to the solution of azidoaniline (60 mg, 0.4473 mmol) in anhydrous THF (2.0 mL). Dark yellow solution was cooled in ice cold water bath and freshly distilled methacryloyl chloride (65 µL, 0.67 mmol, 1.5 equiv)[2] was added dropwise. Reaction mixture was slowly allowed to reach RT and stirred overnight (16 h). Crude reaction mixture (dark brown-red solution) was diluted with EtOAc (25 mL), washed with saturated $KHCO_3$ solution (2 x 5 mL) and mQ water. Organic phase was dried (Na_2SO_4), filtered, concentrated in vacuum and the residue was purified by chromatography (EtOAc in PE, 10% to 25%) to afford *N*-(4-azido)-2-methylacrylamide (Az-MAAm, 83 mg, 92 %) as a brownish solid. ^1H NMR ($CDCl_3$, 600

MHz, δ): 7.56 (d, 2H, J_{vic} = 8.5, 2xH_{ortho}), 7.48 (brd s, 1H, NH), 7.00 (d, 2H, J_{vic} = 8.5, 2xH_{meta}), 5.79 (s, 1H, =CH_2 *cis*), 5.47 (s, 1H, =CH_2 *trans*), 2.06 (s, 3H, CH_3–C=C) ppm [7]. A purified product gave positive colorimetric ninhydrin TLC test [8].

3.1.2 Synthesis of 2-((7-nitrobenzofuraz-4-yl)amino)ethyl methacrylate (NBD-MA)

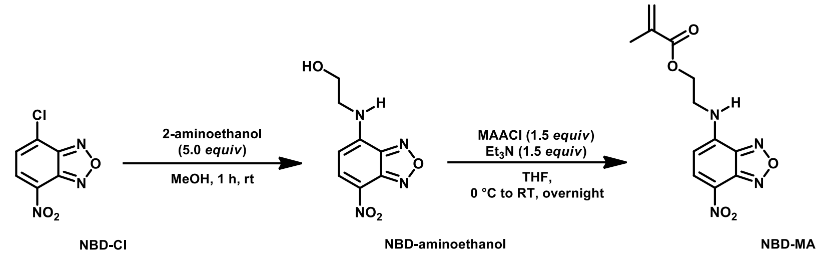

1st step: 2-Aminoethanol (450 μL, 7.50 mmol, 5.0 equiv) was added to the clear yellow solution of 4-chloro-7-nitro-2,1,3-benzoxadiazole (NBD-Cl, 300 mg, 1.50 mmol) in methanol (7.50 mL). Reaction mixture became dark red-brown and was stirred at room temperature for an hour. Reaction mixtures were concentrated, and the residue was purified by chromatography (methanol in chloroform, 0% to 5%) to NBD-aminoethanol as a dark orange solid (283 mg, 84%).

2nd step: Triethylamine (325 μL, 2.34 mmol, 1.5 equiv) was added to the solution of NBD-aminoethanol (350 mg, 1.56 mmol) in anhydrous THF (10.0 mL). Dark red-brown solution was cooled in ice cold water bath and freshly distilled methacryloyl chloride (230 μL, 2.34 mmol, 1.5 equiv)[2] was added dropwise. Reaction mixture was slowly allowed to reach RT and stirred overnight (16 h). Crude reaction mixture (dark brown-red solution) was diluted with EtOAc (50 mL), washed with saturated $KHCO_3$ solution (2 x 10 mL) and mQ water. Organic phase was dried (Na_2SO_4), filtered, concentrated in vacuum and residue was purified by chromatography (EtOAc in PE, 10% to 50%) to afford NBD-MA (328 mg, yield 72 %) as a dark orange-brown oil and an unreacted starting material NBD-aminoethanol (70 mg, conversion 80%) as a dark orange-brown solid.

3.1.3 Synthesis of Carboxybetaine (CBMA) Monomers with Methylene (C1) and Pentylene (C5) Spacers

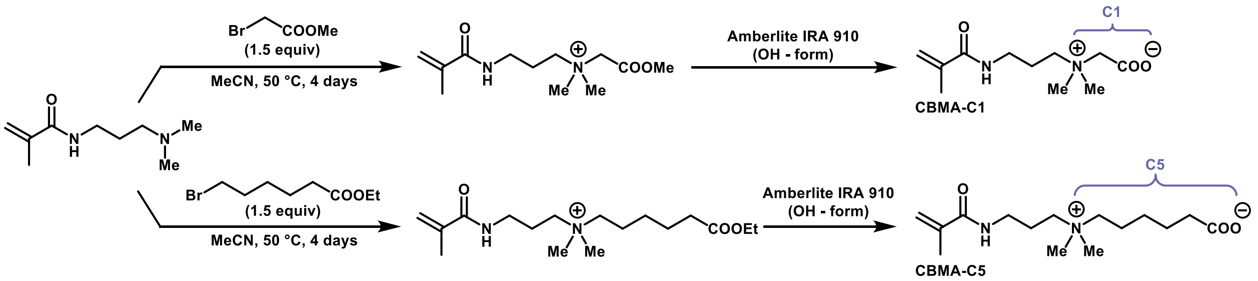

Synthesis of CBMA monomers is based on general procedure employed for synthesis of various carboxybetaines [9–11].

Alkylbromoester (for CBMA-C1: methyl bromoacetate, 7.10 mL, 75 mmol, 1.5 equiv; for CBMA-C5: ethyl 6-bromohexanoate, 13.34 mL, 75 mmol, 1.5 equiv) was added to a solution of *N*-(3-(dimethylamino)propyl)methacrylamide (9.10 mL, 50 mmol) in anhydrous acetonitrile (25 mL). Reaction mixtures were gently heated (50 °C) under nitrogen atmosphere for 4 days. After cooling to RT, oily product was washed with acetone (2 x 50 mL). The upper acetone layer was discarded, and a residue was dissolved in mQ water (100 mL). Aqueous solutions of corresponding esters were recirculated over basic anion exchange resin Amberlite® IRA-910 OH-form column [resin bed volume 150 mL (h 4 cm, r 3.5 cm); flow rate 25 mL/min)] for 2 hours at RT. Solutions were frozen and lyophilized to afford monomers as colorless thick oils (CBMA-C1, 10.34 g, 90%; CBMA-C5, 12.96 g, 91%).

3.2 Synthesis of the Zwitterionic Copolymers

3.2.1 Synthesis of Sulfobetaine Copolymer pSBE, *i.e.* poly[SBE-*co*-Az-*co*-NBD]

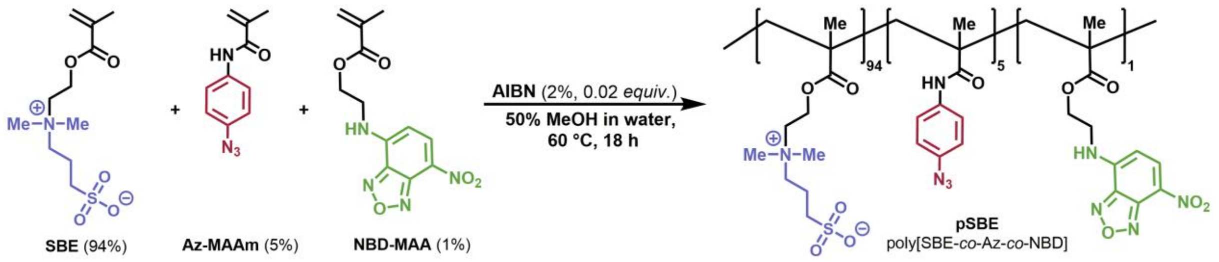

NBD-MA (12 mg, 0.04 mmol, 0.01 equiv, 1% mol) and Az-MAAm (40 mg, 0.20 mmol, 0.01 equiv, 5% mol) dissolved in methanol (5.30 mL) were added to a solution of SBE monomer (1117 mg, 4.00 mmol, 94% mol) in mQ water (5.30 mL) [ΣV = 10.6 mL; c = 0.40 mmol/mL, 10% w) in a Schlenk flask equipped with a magnetic stirrer bar. The mixture was sonicated for a minute at RT in an ultrasound water bath. AIBN (400 μL, 0.08 mmol, 2% mol; 0.2 M solution in toluene) was added. Stirred reaction mixture was bubbled with nitrogen gas for 10 minutes. Sealed flasks were placed on preheated oil bath and the mixture was stirred for 18 h at 60 °C. After 18 h at 60 °C, no precipitation was observed, only light emulsion formation took place after cooling to RT. Reaction mixture was dissolved in mQ water and dialyzed against mQ water for 4 days. The dialysate was filtered (0.2 μm), frozen, and lyophilized to afford a yellow-dark orange foam (650 mg, 56%).

NOTE 1: For UV-Vis analytical purposes, fluorescently non-labelled bipolymer bipoly[SBE-*co*-Az] was synthetized in parallel. Bipolymer was isolated as white-slightly beige foam (730 mg, 63%).

NOTE 2: For evaluation of polymer induced hemolysis bipoly[SBE-*co*-NBD], *i.e.* bipolymer without arylazide moiety, was synthetized under similar experimental conditions and isolated as orange foam (680 mg, 59%).

3.2.2 Synthesis of Carboxybetaine Copolymer pCBMA-C1, *i.e.* poly[CBMA-C1-*co*-Az-*co*-NBD]

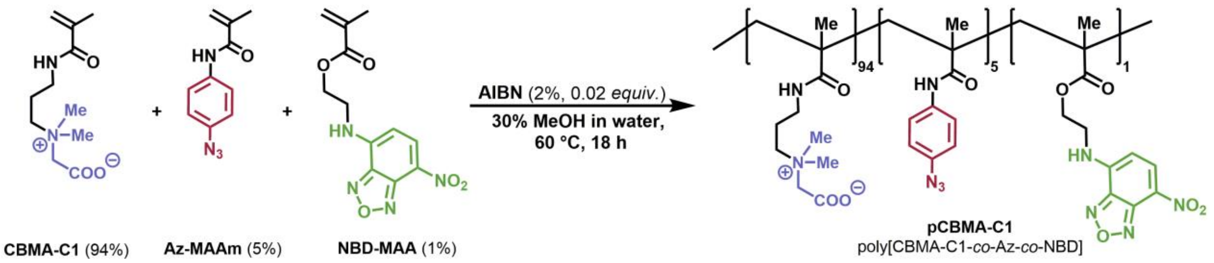

CBMA-C1 (2.15 g, 9.40 mmol, 94% mol) dissolved in mQ water (10.0 mL) was added to a solution of NBD-MA (29 mg, 0.10 mmol, 0.01 equiv, 1% mol) and Az-MAAm (100 mg, 0.50 mmol, 0.05 equiv, 5% mol) in methanol (4.25 mL) [ΣV = 14.25 mL; c = 0.70 mmol/mL, 13.8% w] in a Schlenk flask equipped with a magnetic stirrer bar. Reaction mixture was vigorously stirred overnight at RT for complete dissolution of the reagents. AIBN (1.0 mL, 0.20 mmol, 2% mol; 0.2 M solution in toluene) was added and the stirred reaction mixture (very light gel) was bubbled with nitrogen gas for 15 minutes. Sealed flasks were placed on preheated oil bath and the mixture was stirred for 18 h at 60 °C. Formation of light gel (without solid precipitation) was observed after cooling to RT. The reaction mixture was precipitated to ice cold diethyl ether (200 mL) with the aid of water (≈3 mL). The resulting emulsion was vigorously stirred 1 h at RT and left overnight at -20 °C. Phase separation took place (oil-in-ether), the upper etheric layer was discarded, and dark oil was dissolved in mQ water and dialyzed against mQ water for 4 days. The dialysate was filtered (0.45 μm), frozen, and lyophilized to afford a yellow-brownish glassy foam (0.86 g, 38%).

NOTE 1: For the FTIR study, a sample of the purified and dry pCBMA-C1 was dissolved in 0.1 N HCl and dialyzed against 0.1 N HCl overnight. The dialysate was frozen and lyophilized to afford a yellow glassy foam of the protonated form of pCBMA-C1.

NOTE 2: For evaluation of polymer induced hemolysis, homopoly[CBMA-C1], *i.e.* homopolymer without arylazide and fluorescent moieties, was synthetized under similar experimental conditions and isolated as white-colorless glassy foam (0.47 g, 91%).

3.2.3 Synthesis of Carboxybetaine Copolymer pCBMA-C5, *i.e.* poly[CBMA-C5-*co*-Az-*co*-NBD]

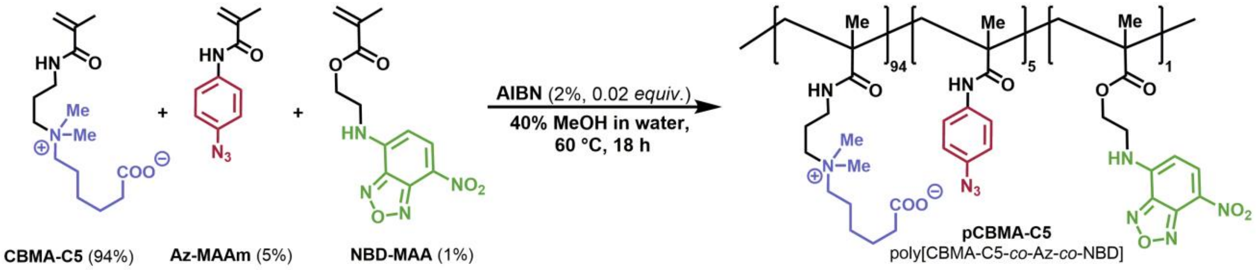

CBMA-C5 (2.67 g, 9.40 mmol, 94% mol) dissolved in mQ water (12.0 mL) was added to a solution of NBD-MA (29 mg, 0.10 mmol, 0.01 equiv, 1% mol) and Az-MAAm (100 mg, 0.50 mmol, 0.05 equiv, 5% mol) in methanol (8.0 mL) [ΣV = 20.0 mL; c = 0.50 mmol/mL, 12.3% w] in a Schlenk flask equipped with a magnetic stirrer bar. Reaction mixture was vigorously stirred overnight at RT for complete dissolution of the reagents. AIBN (1.0 mL, 0.20 mmol, 2% mol; 0.2 M solution in toluene) was added and stirred reaction mixture (light gel) was bubbled with nitrogen gas for 15 minutes. Sealed flasks were placed on preheated oil bath and the mixture was stirred for 18 h at 60 °C. Formation of gel (without solid precipitation) was observed after cooling to RT. Reaction mixture was precipitated to ice cold diethyl ether (200 mL) with aid of water (≈5 mL). Formed emulsion was vigorously stirred for an hour at RT and left overnight at a freezer (-20 °C). Phase separation took place (oil-in-ether), upper etheric layer was discarded, and dark oil was dissolved in mQ water and dialyzed against mQ water for 4 days. The dialysate (light hydrogel) was not suitable for filtration. A product was frozen and lyophilized to afford a yellow-brownish glassy foam (1.20 g, 43%).

NOTE 1: For the FTIR study, a sample of the purified and dry pCBMA-C5 was dissolved in 0.1 N HCl and dialyzed against 0.1 N HCl overnight. The dialysate was frozen and lyophilized to afford a yellow glassy foam of the protonated form of pCBMA-C5.

NOTE 2: For evaluation of polymer induced hemolysis, homopoly[CBMA-C5], *i.e.* homopolymer without arylazide and fluorescent moieties, was synthetized under similar experimental conditions and isolated as white-colorless glassy foam (0.48 g, 74%).

4 Quantitative Analysis of 4-Azido Aniline Moiety in poly[SBE-*co*-Az] Bipolymer by UV/Vis Spectrophotometry

Calibration was performed on commercially available 4-azidoaniline hydrochloride at the concentrations ranging from 0.1314 to 0.7383 µmol/mL (22.5 µg/mL to 125 µg/mL) and plotting the absorbance maxima at 286 nm against concentration (Figure S1). Absorbance was measured in triplicates at λ_{max} = 286 nm. All UV measurements were performed in sodium carbonate – sodium bicarbonate (Na_2CO_3-$NaHCO_3$) buffer solution at pH = 10.8 in semi-micro disposable polystyrene UV cuvettes (1.5 mL) at RT on UV/Visible spectrophotometer Ultrospec 3300 pro (Amersham Biosciences, Little Chalfont, UK). Stock solutions were freshly prepared 30 minutes before the measurement and maintained in dark at RT.

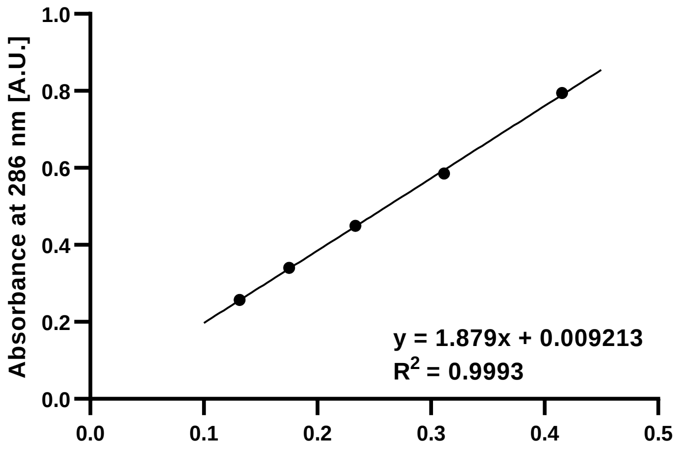

Figure S1. UV calibration curve of 4-azidoaniline hydrochloride in sodium carbonate – sodium bicarbonate (Na_2CO_3-$NaHCO_3$) buffer (pH = 10.8) at 286 nm, n = 3. The error bars are shorter than the height of the symbol and are omitted for clarity.

Sample solution was prepared by dissolving of poly[SBE-*co*-Az] bipolymer in Na_2CO_3-$NaHCO_3$ buffer (pH = 10.8). For the UV analysis solutions with the concentration ranging from 1500 to 844 µg/mL were used. Results are summarized in the Table S1.

Table S1. Calculation of monomers molar ratio

	$c_{poly[SBE-co-Az]}$ [μg/mL]	A [a.u.]	$c_{AzA*HCl}$ [μmol/mL]	$m_{Az-MAAm}$ [μg]	m_{SBE} [μg]	n_{SBE} [μmol]	SBE [%]	Az [%]
**1**	1500	0.558	0.2921	59	1441	5.16	**95%**	**5%**
**2**	1125	0.404	0.2101	42	1083	3.88	**95%**	**5%**
**3**	844	0.298	0.1537	31	813	2.91	**95%**	**5%**

Calculation example:

Table S1, entry 1: absorbance of bipolymer poly[SBE-*co*-Az] solution with concentration 1500 μg/mL is equal to a standard solution of 4-azidoaniline in Na_2CO_3-$NaHCO_3$ buffer (pH = 10.8) with concentration 0.2921 μmol/mL. 1,500 μg of copolymer consist of 5.16 μmol of SBE and 0.2921 μmol of Az-MAAm. Molar ratio of SBE-MAA (FW = 279.35) and Az-MAAm (FW = 202.21) monomers is 95 : 5.

5 4-Azidoaniline Hydrochloride Photolysis (FTIR Kinetic Study)

4-Azidoaniline hydrochloride UV light promoted photolysis was studied by FTIR by decreasing of the intensity of azide peak at 2120 cm^{-1}. 4-Azidoaniline hydrochloride was mixed in a mortar with dry KBr (0.25% w). A pellet was made from 100 mg of the mixture in 10 mm die by applying pressure (4 tons) for 2 min. A pellet was placed in a collar of the pellet holder and set at distance of 20 cm from UV lamp (254 nm, Osram Puritec HNS 15W G13, 210 lux, OSRAM GmbH, Munich, Germany). FTIR spectra were recorded at 0, 1, 2.5, 5, 10, 15, 20, 30, 45 and 60 min (Figure S2).

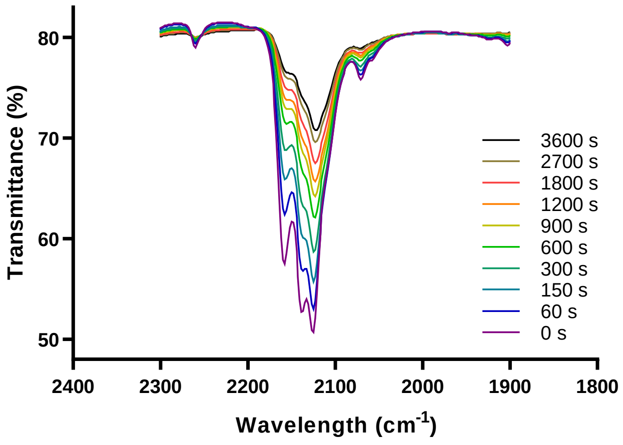

Figure S2. FTIR study of 4-azidoaniline hydrochloride photolysis, decreasing of azide (Ar-N=N^+=N^-) asymmetric stretching vibrational band intensity over time under exposing of UV light.

During the photochemical reaction of 4-azidoaniline hydrochloride, the darkening of the KBr pellet was observed. Color change (from colorless to yellow/brown) was observed after photo-irradiation of the crystal of pure arylazides as well [12]. This observation indicates formation of the triplet nitrene reactive intermediate which undergoes further reactions as e.g. active hydrogen insertion or ring expansion in presence of suitable substrates (Figure S3) [13].

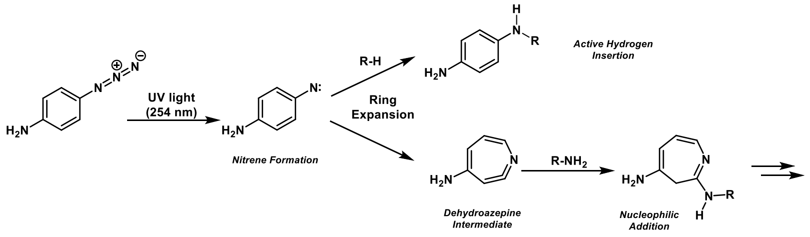

Figure S3. Scheme of photolysis reaction of 4-azidoaniline and expected formation of adducts.

6 Evaluation of Coating Stability

- Static Conditions (batch test): Coated DALI-pSBE (50 mg, approx. 100 μL dry) beads were resuspended in saline (0.9% NaCl, 10 mL) and placed in an overhead stirrer and rotated for 14 days at RT at dark. Supernatant was changed at day: 3, 5, 7, 10, 12 and 14.
- Dynamic Conditions (flow setting): Coated DALI-pSBE beads were placed in a column (adsorbent bed volume 0.5 mL). Solution of bovine serum albumin (BSA, 4%) in PBS (22 mL) was purged through a cartridge by a syringe pump (flow rate 100 μL/min) and 20 fractions (1 mL) were collected (Figure S4).

As a control material for both evaluated conditions, physically pre-coated beads, *i.e.* beads before the exposure to UV light (2nd step, Figure 2 in main text), were employed.

Quantification of Light Intensity

Both types of the beads (+/- UV) were separately placed on one microscopic slide and evaluated by fluorescence microscopy (green FITC emission channel). At least 20 beads per group and three microscopic slides were analyzed (Figure S4). Light intensity was quantified by Corrected Total Particle Fluorescence (CTPF) calculated by the formula: *CTPF = Integrated Density – (Area of Selected Particle × Mean Fluorescence of Background).* Measurements were performed with ImageJ software [14]. CTPF is analogy to total cell fluorescence measurement proposed by Burgess and co-workers [15].

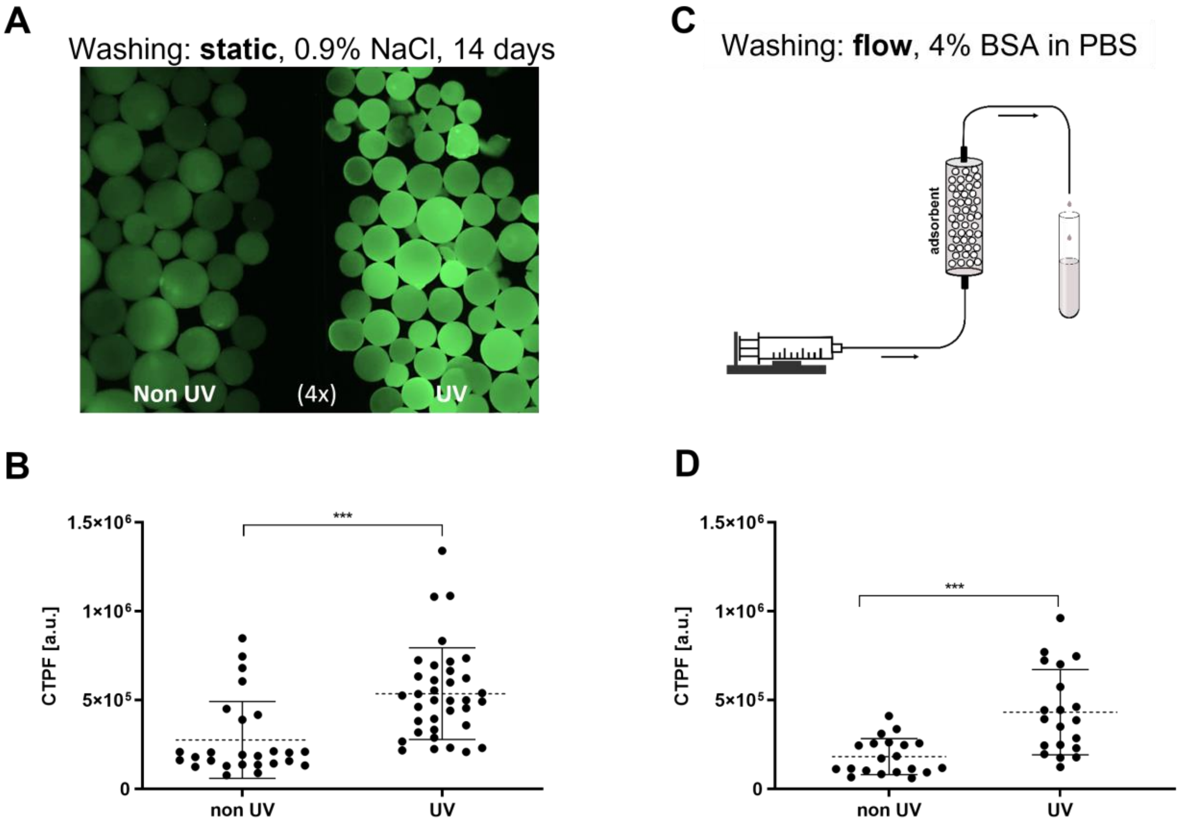

Figure S4. Coating stability: (A) representative fluorescence image of non-UV treated (control) and UV crosslinked DALI-pSBE beads after static washing. (B) CTPF quantification (arbitrary units, a.u.) of image (A) with n = 26 and 36; (C) schematic representation of beads washing under dynamic conditions and (D) CTPF quantification of representative fluorescence image with n = 20 and 20. The scatter plots (B and D) show mean as mean ± SD and variables of significance (***p ≤ 0.001) were calculated by unpaired t-test.

7 Energy-Dispersive X-ray Spectroscopy Analysis

Table S2. Elements in the EDX analysis.

		C	N	O	S
**DALI**	weight [%]	54.18	18.24	27.59	n. d.
	atom [%]	59.85	17.28	22.88	n. d.
**DALI-pSBE**	weight [%]	54.24	17.93	27.77	**0.06**
	atom [%]	59.94	16.99	23.04	0.02
**DALI-pCBMA-C1**	weight [%]	54.14	18.72	27.14	n. d.
	atom [%]	59.78	17.72	22.5	n. d.
**DALI-pCBMA-C5**	weight [%]	55.24	17.72	27.03	n. d.
	atom [%]	60.89	16.75	22.37	n. d.
**CG161c**	weight [%]	**95.26**	**n. d.**	4.78	n. d.
	atom [%]	96.40	n. d.	3.60	n. d.
**CG161c-pSBE**	weight [%]	**78.96**	**8.13**	12.74	**0.16**
	atom [%]	82.63	7.3	10.01	0.06
**CG161c-pCBMA-C1**	weight [%]	**78.62**	**11.94**	9.44	n. d.
	atom [%]	81.94	10.67	7.39	n. d.
**CG161c-pCBMA-C5**	weight [%]	**73.71**	**10.64**	15.64	n. d.
	atom [%]	77.93	9.65	12.42	n. d.

8 Evaluation of Polymer-Induced Hemolysis

Human whole blood anticoagulated with sodium citrate was incubated with adsorbents (10% vol batch test) or solution of polyzwitterions (1.0 mg/mL) for 4 h at 37 °C. NaCl solution (0.9%) was incubated along with the samples as negative control. After incubation, samples and the negative control were centrifuged (2,000 x g, 10 min, RT). Quantification of free hemoglobin (fHb) in plasma was performed by Kahn spectrophotometric method [16] from undiluted plasma (100 µL) in microtiter plates (96 wells). The absorbance A of the supernatant was determined at 578, 562, and 598 nm using a Synergy™ 2 Multi-Mode Microplate Reader (BioTek Instruments, Winooski, VT). fHb (Figure S5) was calculated according to the equation:

$$fHb\ (mg/dL) = (A_{578} \times 155) - (A_{562} \times 86) - (A_{598} \times 69)$$

As a positive control, 1,000 times diluted frozen and thawed whole blood was used.

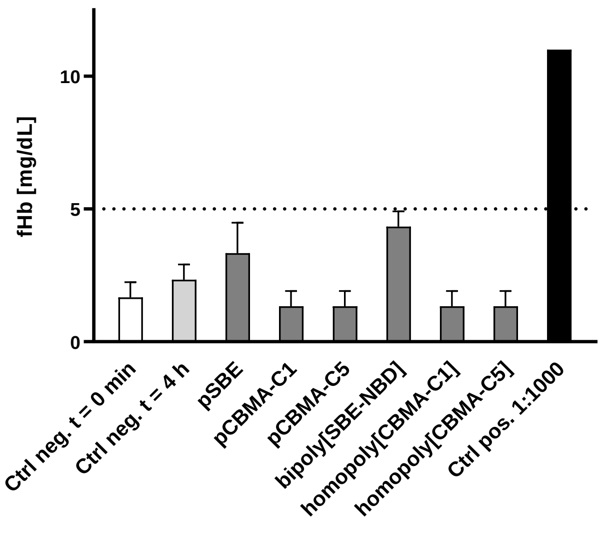

Figure S5. fHg in plasma determined according to Kahn spectrophotometric method after 4 h incubation (37 °C) with +/- tested polyzwitterions in whole blood (sodium citrate) at concentration 1.0 mg/mL. Data are given as mean ± SD, n = 3. Dotted line indicates physiological level of fHb in plasma [3,17].

NOTE 1: No significant hemolysis was observed even at a concentration 10 mg/mL of polyzwitterions in human whole blood (sodium citrate).

NOTE 2: No hemolysis was observed in 10% vol batch test with coated adsorbents.

9 Recirculation of Whole Blood over DALI Columns

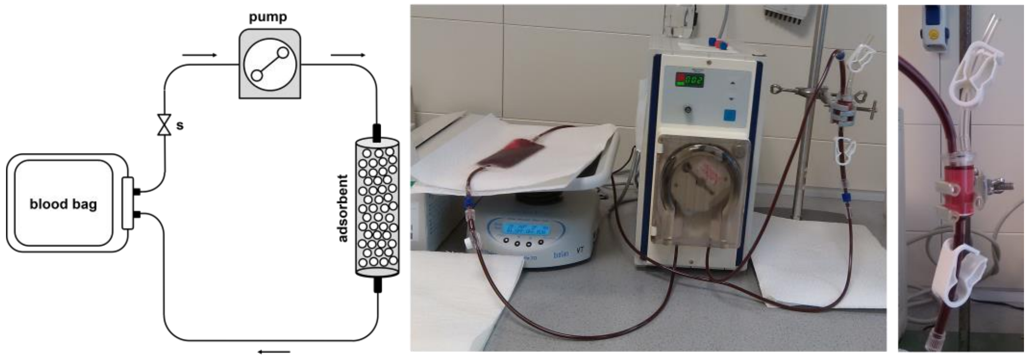

Figure S6. Scheme (left) and photography (right) of adsorption circuit and column detail: Aliquots of 50 mL of whole blood anticoagulated with ACD-A (1:20) and 0.8 IU/mL heparin were recirculated over columns (5 mL) containing (coated *vs.* uncoated) DALI at a flow rate of 1.2 mL/min for 4 h using sterile medical grade tubing sets and a hemodialysis roller pump.

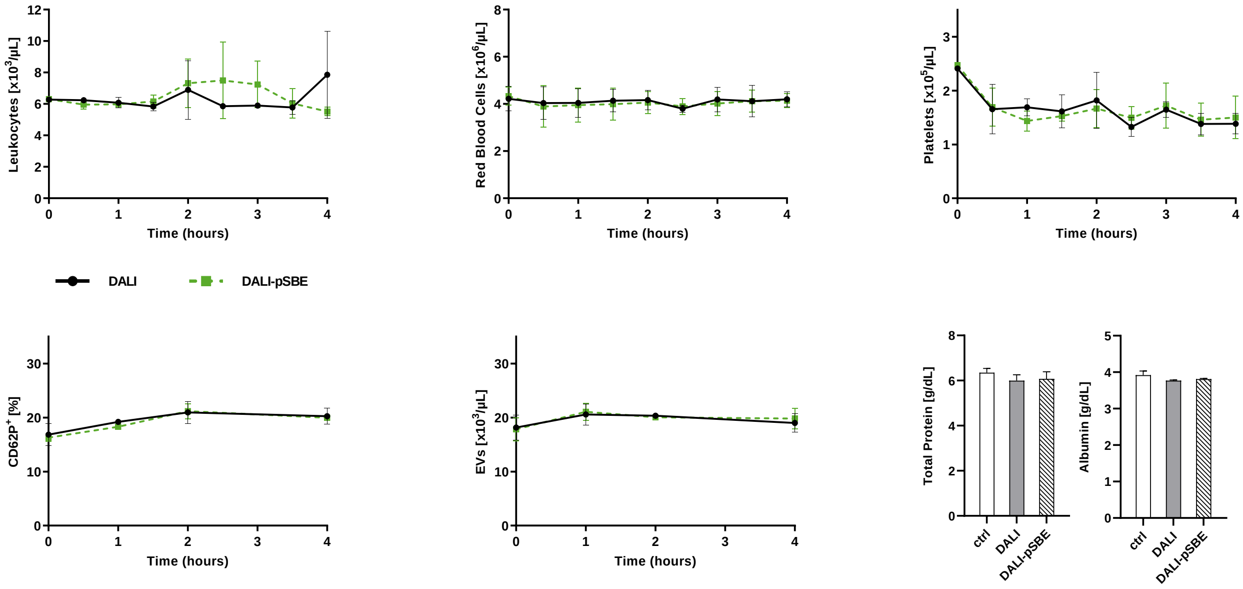

Figure S7. Cell counts, cellular activation, and protein adsorption upon recirculation of human whole blood over DALI columns. Counts of leukocytes, red blood cells and platelets were quantified over time in the flow-through of the adsorbent columns using a blood cell counter as described in the Materials and Methods (section 2.6). Platelet activation using CD62P as platelet activation marker and the release of extracellular vesicles (EVs) were quantified by flow cytometry (SI Methods). Plasma concentration of albumin and total protein at the onset (0 min) and at termination (4h) of the recirculation experiment, ctrl = plasma incubated without adsorbent; data are presented as mean ± SD, n = 3.

10 Scanning Electron Microscopy (SEM) Micrographs of Adsorbents

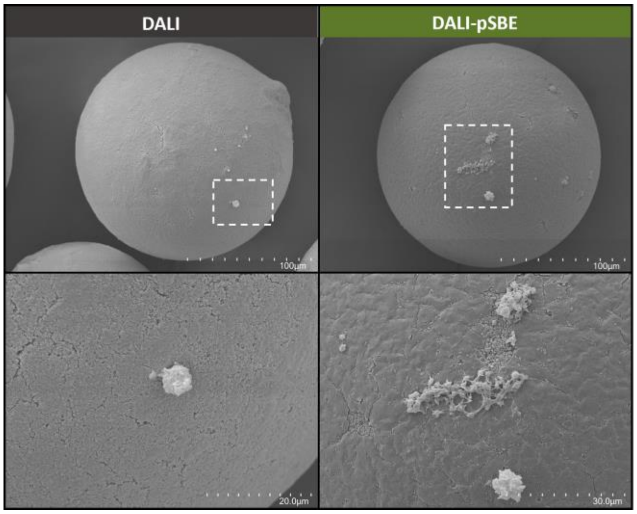

Figure S8. Scanning electron micrographs of DALI adsorbent beads after recirculation of whole blood over adsorbents columns (RT, 4 h). White dotted square indicates zoomed area.

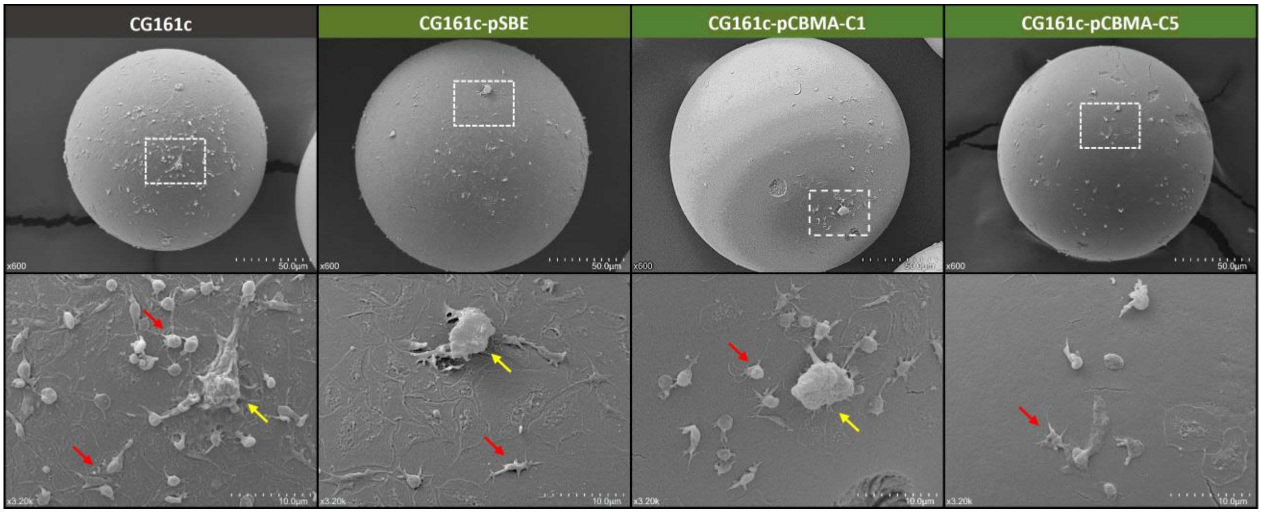

Figure S9. Scanning electron micrographs of CG161c adsorbent beads after incubation with citrated whole blood (37 °C, 60 min). White dotted square indicates zoomed area, red arrows indicate platelets and yellow arrows indicate leukocytes.

11 Tables of the Most Important FTIR Absorption Bands

FTIR Table 1 Frequencies (cm^{-1}) of the most important FTIR absorption bands and proposed assignments of nitrobenzofurazan (NBD) derivatives.

	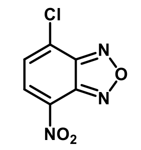 **NBD-Cl** [CAS: 10199-89-0]	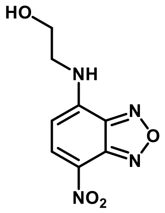 **NBD-aminoethanol**	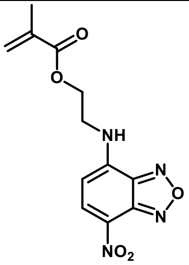 **NBD-MA**
arC-NO_2 as, CC and N=C st	1518	1579	1535
ring deformation	1450	1478	1447
N=C and CC st	1366	1369*	1372*
arC-NO_2 st sy	1326	1299* brd	1334*
N_2O sy st and ip	1050	1066 / 1047	1084
arC-H oop	863	860	863
R′RN-H δ	x	1619	1628
C-N st	x	1284*	1293*
C=O st (ester)	x	x	1716
C=C st (vinylidene)	x	x	1670
C-O st (ester)	x	x	1155
=CH δ oop (vinylidene)	x	x	939

Note: Peaks labeled with asterisk (1280–1380 cm^{-1}, arC-NO_2, C-N and N=C) could be interchanged [18].

FTIR Table 2 Frequencies (cm^{-1}) of the most important FTIR absorption bands and proposed assignments of aryl azide derivatives.

	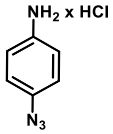 **4-Azidoaniline x HCl** [CAS: 91159-79-4]	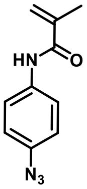 **Az-MAAm**
$-NH_3^+$ st	2826 brd	x
$-N=N^+=N^-$ st as	2120	2112
arC-arC st	1501 brd	1513* brd
$-N=N^+=N^-$ st sy	1294	1287
arC-H δ oop	836 / 820	820 / 803
C=O st (amide I)	x	1659
C=C st (vinylidene)	x	1625
N-C=O st sy (amide II)	x	1513* brd
$-CH_3$ δ (vinylidene)	x	1413
=CH δ oop (vinylidene)	x	929

Note: Bands for arC-arC and C=O stretching of amide II are overlapped to one broad peak (1513 cm^{-1}) label with asterisk. For more details about infrared spectra of organic azides, please refer to Lieber and co-workers [19].

FTIR Table 3 Frequencies (cm^{-1}) of the most important FTIR absorption bands and proposed assignments of sulfobetaine monomer and copolymer.

	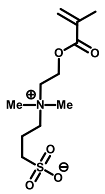 **SBE**	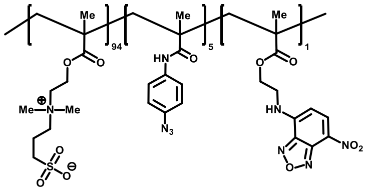 **pSBE**
C=O st (ester)	1716	1723
C=C st (vinylidene)	1637	x
$(CH_3)_2$-N^+ δ as and -CH_2- δ	1484 / 1457	1480 brd
C-N st	1298	
C-O st (ester)	1160	1172 brd
R-SO_3^- st as		
R-SO_3^- st sy	1033	1036
R´R-$N^+(CH_3)_2$ st and δ as	956 / 930*	952 / 931*
=CH δ oop (vinylidene)	930* / 815	x
-N=N^+=N^- st as	x	2121

Note 1: Peaks at 1160-1172 cm^{-1} (C-O, C-N and R-SO_3^- stretching) are overlapping.[9]

Note 2: Peaks for vinylidene =CH (≈ 930 cm^{-1}, out of plane bending) overlap with the asymmetric bending and stretching of -$N^+(CH_3)_2$- moiety.[20]

Note 3: Appearance of water broad stretching vibration absorption band at 3440 cm^{-1}, present in both sulfobetaine spectra, indicates hydrophilicity of employed sulfobetaines. This behavior is expected because simple quaternary ammonium salts, *per se*, are hydrophilic when aliphatic chains are shorter than 5 carbons [20].

Note 4: FTIR spectra of NBD non labelled bipolymer bipoly[SBE-Az] is essentially identical to terpolymer pSBE.

FTIR Table 4 Frequencies (cm^{-1}) of the most important FTIR absorption bands and proposed assignments of carboxybetaine monomers.

	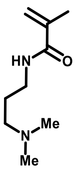 **CAS: 5205-93-6**	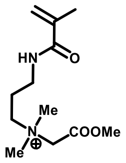 **CBMA-C1-ester**	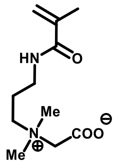 **CBMA-C1-ZW**	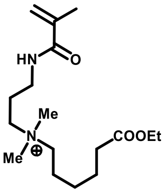 **CBMA-C5-ester**	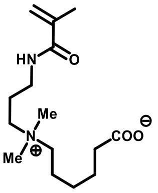 **CBMA-C5-ZW**
C=O st ester	x	1744	x	1725	x
$(COO)^-$ st as	x	x	1616 brd	x	1570 brd
C=O st (amide I)	1655	1656		1658	1656
C=C st (vinylidene)	1614	1615		1616	1614
N-C=O st sy (amide II)	1531	1530	1533	1527	1537
$-CH_3$ δ as (at N) and $-CH_2-$ δ	1455	1446 brd	1454 brd	1453 brd	1487 / 1453
$(COO)^-$ st sy	x	x		x	
$-CH_3$ δ sy (at N)	1375 / 1311	1375 / 1317	1376 / 1323	1373 / 1302	1384 / 1308
C-O st ester	x	1235 / 1212*	x	1212* / 1180	x
C-N st	1217	1212*	1218	1212*	1220
=CH δ oop (vinylidene)	925	940	931	930	929

Note 1: Peaks labeled with asterisk (1212 cm^{-1}, C-O and C-N stretching) are overlapping.

Note 2: Vinylidene=CH out of plane bending (≈ 930 cm^{-1}) overlap with the asymmetric bending and stretching of $-N^+(CH_3)_2-$ moiety [20].

FTIR Table 5 Frequencies (cm^{-1}) of the most important FTIR absorption bands and proposed assignments of carboxybetaine copolymers at acidic and basic pH.

	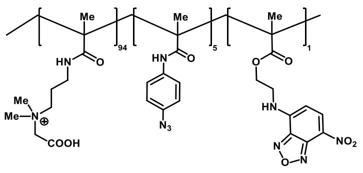 **pCBMA-C1** acidic pH	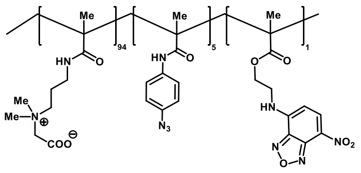 **pCBMA-C1** basic pH	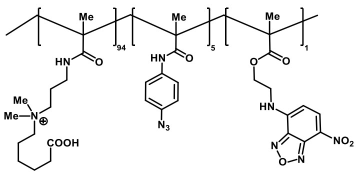 **pCBMA-C5** acidic pH	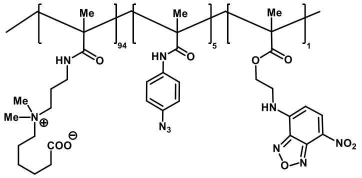 **pCBMA-C5** basic pH
$-N{=}N^{+}{=}N^{-}$ st as	2120	2122	2118	2118
C=O st carboxylic acid	1733	x	1716	x
$(COO)^{-}$ st as	x	1621 brd	x	1567 brd
C=O st (amide I)	1635		1643	1635
N-C=O st sy (amide II)	1527	1531	1527	1525
$(CH_3)_2$-N^{+} δ as and $-CH_2-$ δ	1480 brd	1482 brd	1483 brd	1483 brd
$(COO)^{-}$ st sy	x		x	
$-CH_3$ δ sy (at N)	1393 brd	1386/1326	1392/1371	1386 brd
C-N st	1197 brd	1203	1200 brd	1202

Note 1: For more details about pH induced changes of FTIR spectra of carboxybetaine copolymers please refer to Abraham and co-workers [21] and for influence of the length of the tether group please refer to Weers and co-workers [9].

Note 2: FTIR spectra of homopolymers homopoly[CBMA-C1] and homopoly[CBMA-C5] are essentially identical to terpolymers pCBMA-C1 and pCBMA-C5, except for the lack of the azide peak (≈ 2120 cm^{-1}).

12 Abbreviations

BSA	Bovine Serum Albumin
EtOAc	Ethyl acetate
EtOH	Ethanol
Et_3N	Triethylamine
MA	Methacrylate
MAAm	Methacrylamide
MAA	Methacrylic acid
MAACl	Methacrylic acid chloride (Methacryloyl chloride)
MeCN	Acetonitrile
MeOH	Methanol
mQ water	MilliQ (18.2 MΩ·cm) water at 25 °C
NBD	7-Nitro-1,2,3-benzoxadiazole
PBS	Phosphate-Buffered Saline
PE	Petroleum Ether
THF	Tetrahydrofuran
TLC	Thin-Layer Chromatography

Abbreviation used in FTIR tables

ar	aromatic
al	aliphatic
as	asymmetric
brd	broad signal
δ	deformation vibration
ip	in plane vibration
oop	out of plane vibration
st	stretching vibration
sy	symmetric

13 References

[1] A.R. Hajipour, F. Mohammadsaleh, Synthesis of aryl azides from aryl halides promoted by Cu2O/tetraethylammonium prolinate, Tetrahedron Lett. 55 (2014) 6799–6802. https://doi.org/10.1016/j.tetlet.2014.10.045.

[2] J. Warneke, Z. Wang, M. Zeller, D. Leibfritz, M. Plaumann, V. Azov, Methacryloyl chloride dimers: from structure elucidation to a manifold of chemical transformations, Tetrahedron. 70 (2014) 6515–6521. https://doi.org/10.1016/j.tet.2014.07.019.

[3] ISO:10993-4, Biological evaluation of medical devices — Part 4: Selection of tests for interactions with blood, (2017). https://www.iso.org/obp/ui#iso:std:iso:10993:-4:ed-3:v1:en.

[4] R. Weiss, M. Gröger, S. Rauscher, B. Fendl, T. Eichhorn, M.B. Fischer, A. Spittler, V. Weber, Differential Interaction of Platelet-Derived Extracellular Vesicles with Leukocyte Subsets in Human Whole Blood, Sci. Rep. 8 (2018) 6598. https://doi.org/10.1038/s41598-018-25047-x.

[5] B. Fendl, R. Weiss, T. Eichhorn, I. Linsberger, T. Afonyushkin, F. Puhm, C.J. Binder, M.B. Fischer, V. Weber, Extracellular vesicles are associated with C-reactive protein in sepsis, Sci. Rep. 11 (2021) 6996. https://doi.org/10.1038/s41598-021-86489-4.

[6] S.K. George, L. Lauková, R. Weiss, V. Semak, B. Fendl, V.U. Weiss, S. Steinberger, G. Allmaier, C. Tripisciano, V. Weber, Comparative analysis of platelet-derived extracellular vesicles using flow cytometry and nanoparticle tracking analysis, Int. J. Mol. Sci. 22 (2021) 3839. https://doi.org/10.3390/ijms22083839.

[7] Y. Ito, H. Hasuda, M. Sakuragi, S. Tsuzuki, Surface modification of plastic, glass and titanium by photoimmobilization of polyethylene glycol for antibiofouling., Acta Biomater. 3 (2007) 1024–1032. https://doi.org/10.1016/j.actbio.2007.05.010.

[8] B. Cegielska, K.M. Kacprzak, Simple and Convenient Protocol for Staining of Organic Azides on TLC Plates by Ninhydrin. A New Application of an Old Reagent, Chem. Analityczna. 54 (2009) 807–812.

[9] J.G. Weers, J.F. Rathman, F.U. Axe, C.A. Crichlow, L.D. Foland, D.R. Scheuing, R.J. Wiersema, A.G. Zielske, Effect of the intramolecular charge separation distance on the solution properties of betaines and sulfobetaines, Langmuir. 7 (1991) 854–867. https://doi.org/10.1021/la00053a008.

[10] S. Abraham, L.D. Unsworth, Multi-functional initiator and poly(carboxybetaine methacrylamides) for building biocompatible surfaces using "nitroxide mediated free radical polymerization" strategies, J. Polym. Sci. Part A Polym. Chem. 49 (2011) 1051–1060. https://doi.org/10.1002/pola.24517.

[11] E.E. Kathmann, L.A. White, L. McCormick, Water soluble polymers: 70. Effects of methylene versus propylene spacers in the pH and electrolyte responsiveness of zwitterionic copolymers

incorporating carboxybetaine monomers, Polymer (Guildf). 38 (1997) 879–886. https://doi.org/10.1016/S0032-3861(96)00587-3.

[12] T. Takayama, T. Mitsumori, M. Kawano, A. Sekine, H. Uekusa, Y. Ohashi, T. Sugawara, Direct observation of arylnitrene formation in the photoreaction of arylazide crystals, Acta Crystallogr. Sect. B Struct. Sci. 66 (2010) 639–646. https://doi.org/10.1107/S0108768110036608.

[13] N. Gritsan, M. Platz, Photochemistry of Azides: The Azide/Nitrene Interface, in: Org. Azides, John Wiley & Sons, Ltd, 2010: pp. 311–372. https://doi.org/10.1002/9780470682517.ch11.

[14] C.A. Schneider, W.S. Rasband, K.W. Eliceiri, NIH Image to ImageJ: 25 years of image analysis, Nat. Methods. 9 (2012) 671–675. https://doi.org/10.1038/nmeth.2089.

[15] A. Burgess, S. Vigneron, E. Brioudes, J.-C. Labbé, T. Lorca, A. Castro, Loss of human Greatwall results in G2 arrest and multiple mitotic defects due to deregulation of the cyclin B-Cdc2/PP2A balance, Proc. Natl. Acad. Sci. 107 (2010) 12564–12569. https://doi.org/10.1073/pnas.0914191107.

[16] S.E. Kahn, B.F. Watkins, E.W.J. Bermes, An evaluation of a spectrophotometric scanning technique for measurement of plasma hemoglobin., Ann. Clin. Lab. Sci. 11 (1981) 126–131.

[17] G. Lippi, Systematic Assessment of the Hemolysis Index: Pros and Cons (Chapter 6), in: G.S. Makowski (Ed.), Adv. Clin. Cemistry, Elsevier, 2015: pp. 157–170. https://doi.org/https://doi.org/10.1016/bs.acc.2015.05.002.

[18] M. Kurt, P.C. Babu, N. Sundaraganesan, M. Cinar, M. Karabacak, Molecular structure, vibrational, UV and NBO analysis of 4-chloro-7-nitrobenzofurazan by DFT calculations, Spectrochim. Acta - Part A Mol. Biomol. Spectrosc. 79 (2011) 1162–1170. https://doi.org/10.1016/j.saa.2011.04.037.

[19] E. Lieber, C.N.R. Rao, T.S. Chao, C.W.W. Hoffman, Infrared Spectra of Organic Azides, Anal. Chem. 29 (1957) 916–918. https://doi.org/10.1021/ac60126a016.

[20] J.D. Anastassopoulou, Mass and FT-IR Spectra of Quaternary Ammonium Surfactants, in: E. Rizzarelli, T. Theophanides (Eds.), Chem. Prop. Biomol. Syst., Springer Netherlands, Dordrecht, 1991: pp. 1–9. https://doi.org/10.1007/978-94-011-3620-4_1.

[21] S. Abraham, A. So, L.D. Unsworth, Poly(carboxybetaine methacrylamide) Modified Nanoparticles: a Model System for Studying the Effect of Chain Chemistry on Film Properties, Adsorbed Protein Conformation and Clot Formation Kinetics., Biomacromolecules. 12 (2011) 3567–3580. https://doi.org/10.1021/bm200778u.
